# Improvement of Blood Plasmalogens and Clinical Symptoms in Parkinson's Disease by Oral Administration of Ether Phospholipids: A Preliminary Report

**DOI:** 10.1155/2020/2671070

**Published:** 2020-02-19

**Authors:** Shiro Mawatari, Shinji Ohara, Yoshihide Taniwaki, Yoshio Tsuboi, Toru Maruyama, Takehiko Fujino

**Affiliations:** ^1^Institute of Rheological Functions of Food, 2241-1 Kubara, Hisayama-cho, Kasuya-gun, Fukuoka 811-2501, Japan; ^2^Department of Neurosurgery, Fukuoka Sanno Hospital, 3-6-45 Momochihama, Sawara-ku, Fukuoka 814-0001, Japan; ^3^Department of Neurology, Fukuoka Sanno Hospital, 3-6-45 Momochihama, Sawara-ku, Fukuoka 814-0001, Japan; ^4^Department of Neurology, School of Medicine, Fukuoka University, 7-45-1 Nanakuma, Johnan-ku, Fukuoka 814-0133, Japan; ^5^Department of Medicine and Biosystemic Science, Kyushu University Faculty of Medicine, 3-1-1 Maidashi, Higashi-ku, Fukuoka 812-8582, Japan; ^6^BOOCS Clinic, 6F 6-18 Tenyamachi, Hakata-ku, Fukuoka 812-0025, Japan

## Abstract

*Introduction*. Parkinson's disease (PD) is the second most common neurodegenerative disease after Alzheimer's disease (AD). With the ageing of population, the frequency of PD is expected to increase dramatically in the coming decades. L-DOPA (1,3,4-dihydroxyalanine) is the most effective drug in the symptomatic treatment of PD. Nonmotor symptoms in PD include sleep problems, depression, and dementia, which are not adequately controlled with dopaminergic therapy. Here, we report the efficacy of oral administration of scallop-derived ether phospholipids to some nonmotor symptoms of PD.

**Methods:**

Ten (10) patients received oral administration of 1 mg/day of purified ether phospholipids derived from scallop for 24 weeks. Clinical symptoms and blood tests were checked at 0, 4, 12, 24, and 28 weeks. The blood levels of plasmalogens in patients with PD were compared with those of 39 age-matched normal controls.

**Results:**

Initial levels of plasma ethanolamine ether phospholipids in PD and ethanolamine plasmalogen of erythrocyte from PD were lower than those of age-matched normal controls. Oral administration of 1 mg/day of the purified ether phospholipids increased plasma ether phospholipids in PD and increased the relative composition of ether phospholipids of erythrocyte membrane in PD. The levels of ether phospholipids in peripheral blood reached to almost normal levels after 24 weeks. Furthermore, some clinical symptoms of PD improved concomitantly.

**Conclusion:**

1 mg/day of oral administration of purified ether phospholipids derived from scallop can increase ether phospholipids in peripheral blood and concomitantly improve some clinical symptoms of PD.

## 1. Introduction

Parkinson's disease (PD) is a neurodegenerative disorder characterized by cytoplasmic fibrillary aggregates of *α*-synuclein (Lewy bodies) and associated loss of dopaminergic cells in the substantia nigra [1–3]. PD affects as many as 1-2% of persons aged 60 years and older [1]. With the ageing of population, the frequency of PD is expected to increase dramatically in the coming decades [1–3]. The hallmark symptoms of PD are resting tremor, rigidity, bradykinesia, and postural instability [1–3]. Those symptoms are related to dopamine deficiency [1–3]. Current therapies treat these symptoms by replacing or boosting existing DA. L-DOPA (1,3,4-dihydroxyalanine) is the most effective drug in the symptomatic treatment of PD, but chronic use of l-DOPA leads to l-DOPA-induced dyskinesia [4]. Nonmotor symptoms of PD include sleep problems, dysautonomia, depression, and dementia [1, 5–9], which are not adequately controlled with dopaminergic therapies and greatly influence the quality of life.

Ether phospholipids represent a specific subclass of glycerophospholipids characterized by the presence of an ether bond at the sn-1 position of glycerol backbone [10–13]. There are two types of ether bonds in ether phospholipids: alkyl bond and alkenyl bond. Glycerophospholipids with alkenyl bond (vinyl ether bonds) are called plasmalogens [10–13]. Plasmalogens are not only a structural component of cell membranes and a reservoir for secondary messengers, but may also be involved in membrane fusion, ion transport, cholesterol efflux, and antioxidation in cell membrane [10–14].

We previously reported efficacy of oral administration of purified ether phospholipids derived from scallop to cognitive functions in patients with mild Alzheimer's disease (AD) and mild cognitive impairment (MCI) by a multicenter, randomized, double-blind, placebo-controlled trial [15, 16]. In that study, 140 patients with AD received 1 mg/day of ether phospholipids derived from scallop for 24 weeks, and no serious adverse events have been observed [15].

Previously, we observed that many patients with PD showed decreased levels of ether phospholipids in peripheral blood as compared to those of age-matched normal controls. Therefore, we carried out a trial, in which the purified ether phospholipids derived from scallop were orally given to 10 patients with PD for 24 weeks. The oral administration of the ether phospholipids increased ether phospholipids in peripheral blood and showed a concomitant improvement in some clinical symptoms of PD.

## 2. Materials and Methods

### 2.1. Ingredient of Capsule

One soft capsule contained 0.28 mg of ethanolamine ether phospholipids (ePE), 0.22 mg of choline ether phospholipid (ePC), 0.08 mg of cholesterol, and 0.07 mg of ceramide aminoethylphosphonate (CAEP). Lipid composition of the capsule and fatty acid composition of each ether phospholipid are listed in Tables 1 and 2. It was confirmed by acid (HCl) hydrolysis of the purified ether phospholipids from scallop that 93.2% of ePE is alkenyl acyl phospholipid (plasmalogens), but 95.2% of ePC was alkyl acyl phospholipid.

### 2.2. Study Participants

Fourteen (14) patients with PD were recruited, of which 7 patients had a history of deep brain stimulation (DBS) therapy. Diagnosis of PD was done by clinical symptoms. Some baseline characteristics of the patients are shown in Table 3. Patients taking anti-Parkinson drugs (including dopamine agonists) had no changes in the regimen during the previous one month and kept unchanged during the trial period. The patients ingested two capsules a day, usually after breakfast and after supper. Thus, each patient took 1 mg/day of ether phospholipids (ePE + ePC). The patients received the capsules for 24 weeks and 4 weeks of observation without administration of the capsules (posttreatment period). Clinical symptoms of patients and blood test (including assays of plasma and erythrocyte ether phospholipids) were checked at 0, 4, 12, 24, and 28 weeks of treatment. Written informed consent was obtained from either patients or their caregivers. Finally, 10 patients completed the trial (Table 3). The blood levels of plasmalogens in patients with PD were compared with those of 39 age-matched normal controls who had no evidence of medicated diseases or cognitive decline (Table 3). Written informed consent was obtained from each normal volunteer.

The study was approved by the Institutional Review Boards of Fukuoka University School of Medicine and the Review Board of BOOCS Clinic (Fukuoka, Japan). The study was implemented in compliance with the Declaration of Helsinki.

### 2.3. Preparation of Plasma and Erythrocytes

Venous blood of the subjects who had fasted overnight was drawn into a tube containing heparin. The blood was cooled in an ice bath and kept in the refrigerator for 48 h at maximum. After centrifugation at 1000 ×g for 5 min at 4°C, plasma was kept at −30°C until measurement. The packed erythrocytes were washed three times with physiological saline solution, and residual plasma and buffy coats were removed at each washing. The washed erythrocytes were lysed in 40 vol. of hypotonic buffer (10 mM Tris-HCl, pH 7.4) and were centrifuged at 25000 ×g for 20 min at 4°C. This procedure was repeated four times for removing hemoglobin, and the erythrocyte membranes (white in color) were obtained [17]. The membranes were kept at −80°C until use.

### 2.4. Measurements of Ether Phospholipids and Sphingomyelin in Plasma

Measurement of ether phospholipids and sphingomyelin (SM) in plasma was done by our method which were described recently [18]. Briefly, phospholipase A_1_ (PLA1) hydrolyzes ester (acyl) bond at the sn-1 position of glycerophospholipids, but PLA1 does not act on ether bond at the sn-1 position. Diacyl phospholipids completely disappeared after treatment of plasma with PLA1, but ether phospholipids remain intact [18]. After the treatment of plasma with PLA1, lipids were extracted with hexane/isopropanol method (3 : 2, v/v) [19] and were measured by LC-ESI-MS method [18]. The PLA1 treatment of plasma did not hydrolyze sphingomyelin (SM) as well as ether phospholipids; therefore, we also quantified SM in parallel with ethanolamine ether phospholipid (ePE) and choline ether phospholipids (ePC). Selected ion monitoring (SIM) was used with ESI detection of ePE, ePC, and SM [18].

### 2.5. Assay of Relative Composition of Phospholipids in Erythrocyte Membrane

Lipids from erythrocyte membranes were extracted with chloroform/methanol (1 : 2, v/v) method [20], and total lipids were dried under nitrogen gas. After the lipids were resuspended in hexane/isopropanol (3 : 2, v/v), phospholipid composition of erythrocyte membrane was measured by HPLC-evaporative light scattering detector (ELSD). The HPLC method can detect ether phospholipids (ePE and ePC) together with all the other phospholipids usually found in cell membrane by a single run of chromatography, but it does not differentiate plasmalogen from alkyl acyl phospholipid [21].

### 2.6. Parkinson's Disease Questionnaire-39 (PDQ-39)

PDQ-39 is a 39-item self-report questionnaire which assesses PD-specific health-related quality of life [22, 23]. We used PDQ-39 for monitoring health status on physical, mental, and social domains of patients with PD. PDQ-39 was checked at 0, 4, 12, 24, and 28 weeks of the treatment.

### 2.7. Statistical Analysis

Data were analyzed by using unpaired *t*-test for between-group differences and paired *t*-test for the within-group differences. Statistical significance was declared when two-sided *p* < 0.05.

## 3. Results

### 3.1. Ether Phospholipids and SM in Plasma

Our previous study confirmed by acid hydrolysis of lipids after PLA1 treatment that chromatographic peaks of ether phospholipids in human plasma contained some alkyl acyl phospholipids in addition to alkenyl acyl phospholipids (plasmalogens) [18]; therefore, we termed the chromatographic peaks in human plasma as ether phospholipids (ePE and ePC) instead of plasmalogens (plsPE and plsPC).

**Figure 1:**
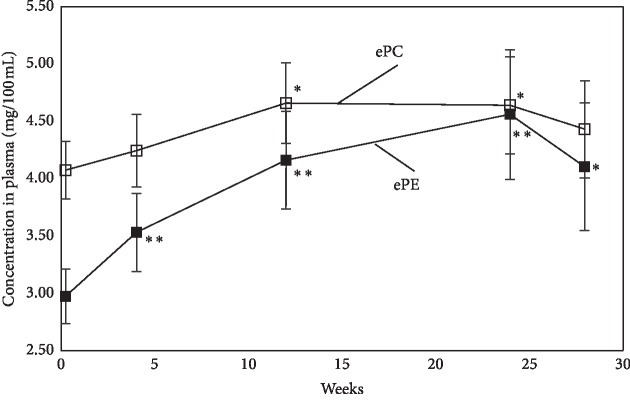
Increases of plasma ethanolamine ether phospholipid (ePE) and plasma choline ether phospholipid (ePC) after oral administration of purified ether phospholipid derived from scallop. Last 4 weeks (24 to 28 weeks) was the observation period without administration of capsules. Differences from immediate before trial; ^*∗∗*^*p* < 0.01, ^*∗*^*p* < 0.05. Error bars indicate standard error.

At the beginning of oral administration of plasmalogens, mean level of plasma ePE in PD was significantly low as compared to those of normal elderly controls. However, the level of plasma ePE increased rapidly after the ingestion of the capsules (Figure 1), and it reached almost the normal level after 12 weeks and the levels were maintained until 24 weeks (Figure 1). Similar temporal changes were observed for ePC and SM levels. After the administration of the capsules was stopped at 24 weeks, the levels of plasma ePE showed a tendency to decrease (Figure 1).

### 3.2. Ethanolamine Plasmalogen in Erythrocyte Membrane

As ether phospholipid, only ethanolamine ether phospholipid was detected in human erythrocyte membrane [21]. Acid hydrolysis of the total lipid from erythrocyte membrane confirmed that the ether lipid in human erythrocyte membrane was ethanolamine plasmalogen (plsPE) [21].

**Figure 2:**
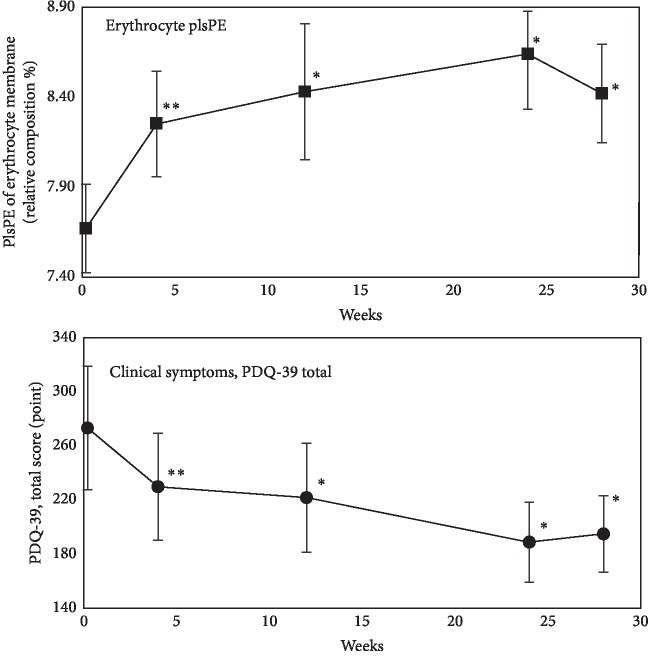
Increase of relative composition of ethanolamine plasmalogen (plsPE) of erythrocyte membrane and improvement of clinical symptoms of PD (PDQ-39 total) after oral administration of purified ether phospholipid derived from scallop. Last 4 weeks (24 to 28 weeks) was the observation period without administration of capsules. Differences from immediate before trial; ^*∗∗*^*p* < 0.01, ^*∗*^*p* < 0.05.

Initially, the relative composition of plsPE in erythrocyte membrane from PD was significantly low as compared to that of normal elderly (Table 4). Relative composition of PC was also low in PD, and the decreases of plsPE and PC were replaced by increase of SM in PD (Table 4). PE and PS were not changed in PD (Table 3). The ingestion of the capsules containing purified ether phospholipids increased the relative composition of plasmalogen (plsPE) of erythrocyte membrane rapidly (Figure 2) and the phospholipid composition of erythrocyte reached almost the normal levels after 24 weeks (Table 4, Figure 2). After the administration of the capsules was stopped at 24 weeks, the levels of plasma ePE showed a tendency to decrease (Figure 2).

### 3.3. Efficacy of Oral Administration of Ether Phospholipids to Clinical Symptoms

The oral administration of the ether phospholipids improved some clinical symptoms of PD concomitantly with increase of ether phospholipids in peripheral blood (Figure 2 and Table 5). The improvement of clinical symptoms was almost in parallel with the increase of erythrocyte plsPE (Figure 2).

## 4. Discussion

The present study showed increase of ether phospholipids in peripheral blood (plasma and erythrocyte membrane) after ingestion of purified ether phospholipids derived from scallop. The ether phospholipid in the erythrocyte membrane is confirmed to be ethanolamine plasmalogen [21]. Plasmalogens are found in almost all mammalian tissues and constitute about 18–20% of the total phospholipids in cell membranes [10, 11]. Predominant plasmalogens in mammalian tissues are ethanolamine plasmalogen (plsPE) and choline plasmalogen (plsPC) [10–12]. It is reported that plasmalogens are abundant in the brain, retina, leukocytes (immune cells), sperm, heart, and skeletal muscle in mammals [10, 11]. Vinyl ether bond at the sn-1 position makes plasmalogens more susceptible to oxidative stress than corresponding ester-bonded glycerophospholipids [11–15]. Therefore, plasmalogens may act as antioxidation and protect cells from oxidative stress [13, 14].

There are indications that high oxidative stress may present in peripheral blood of PD [24]. The increase of oxidized form of coenzyme Q10 in the plasma of PD may indicate elevated systemic oxidative stress in PD [25]. Actually, Dragonas et al. [26] reported decreased plasma plasmalogens as a marker of increased systemic oxidative stress in PD. They measured plasma plasmalogen by transesterification of plasma phospholipids and plasmalogen-derived dimethyl acetal (DMA) was measured with gas chromatography. Presence of neuroinflammation in PD [27] and *α*-synuclein dimerization in erythrocyte in PD has been indicated [28]. The decrease of ether phospholipids in peripheral blood of PD (Table 3) may be partly due to high oxidative stress in PD.

The present trial showed that oral administration of purified ether phospholipids derived from scallop increased plasma and erythrocyte ether phospholipids in PD. Furthermore, some clinical symptoms of PD were improved concomitantly. Beneficial effects of the same ether phospholipids ingestion were also observed in patients with AD [15]. The oral ingestion of purified ether phospholipids derived from scallop improved some cognitive functions of AD [15]. Effectiveness of oral administration of plasmalogen to cognitive functions was also reported in animal models of AD [29].

Physiological mechanisms of the efficacy of such a small amount (1 mg/day) of ether phospholipid to AD and PD are not clear. One hypothesis is that newly administered plasmalogens and/or ether phospholipids may work through some receptors on cell membrane like hormones. Lipid rafts, cholesterol, and SM-rich microdomains on the cell membrane are considered to be associated with cell signaling [30]. There are some reports that lipid rafts are rich in plasmalogens [31]. G-protein-coupled receptors (GPCR) are also localized to lipid rafts and are activated by plasmalogens [32, 33]. These may indicate the possibility that plasmalogens and/or ether phospholipids work as a ligand of GPCR at the lipid rafts.

On the other hand, there have been many reports that docosahexaenoic acid (DHA) and eicosapentaenoic acid (EPA) relate closely to brain functions [34, 35]. Scallop-derived ether phospholipids used in the present study consist of high amounts of DHA and EPA (Table 2); therefore, it is possible that these omega-3 polyunsaturated fatty acids of the ether phospholipids may be effective for improvement of some clinical symptoms of PD. Some studies suggest that DHA in the form of phospholipids passes through the blood-brain barrier approximately ten times more efficiently than in the form of free fatty acid [36]. Moreover, it is recently reported that DHA esterified with lysophospholipids is better incorporated into the brain than its nonesterified form [37, 38]. It is also reported that the uridine and DHA containing diet prevented rotenone-induced motor and gastrointestinal dysfunctions in rotenone-induced PD in mice [39].

The capsule used in the present study contains alkyl acyl choline phospholipid (ePC) and CAEP in addition to ethanolamine plasmalogen (ePE) (Table 1). It is possible that alkyl choline phospholipid (ePC) was an effective ingredient rather than ethanolamine plasmalogen (ePE), because alkylphospholipid analogs constitute a family of synthetic antitumor compounds that target cell membrane [40]. Alkylphospholipids are easily inserted into the outer leaflet of the plasma membrane [41]. On the other hand, the capsule contains ceramide. Therefore, it is also probable that CAEP is an effective ingredient of the capsule [42]. Anyway, physiological mechanisms of the efficacy of the purified ether phospholipid derived from scallop remained to be elucidated.

Effectiveness of plasmalogens to symptoms of PD was suggested by animal models of PD. It has been reported that plasmalogen precursor analog treatment reduced levodopa-induced dyskinesia in parkinsonian monkeys [43], and the same group also reported that plasmalogen augmentation with a plasmalogen precursor reversed striatal dopamine loss in 1-methyl-4-phenyl-1,2,3,6-tetrahydropyridine- (MPTP-) treated mice [44].

In summary, ethanolamine ether phospholipids (ePE) in plasma from PD and relative composition of ethanolamine plasmalogen (plsPE) of erythrocyte membrane in PD were significantly low as compared to those of age-matched normal controls. Oral administration of purified ether phospholipids derived from scallop for 24 weeks increased plasma ePE and erythrocyte plsPE to almost the normal levels and concomitantly improved some clinical symptoms of patients with PD. The results indicate the efficacy of oral administration of purified ether phospholipids derived from scallop to some nonmotor symptoms of PD.

## Figures and Tables

**Table 1:** Lipid composition of purified ether phospholipids from scallop.

Lipid	(mg/g)
Cholesterol (Chol)	11.5
Ethanolamine ether phospholipid (ePE)	42.6
Choline ether phospholipid (ePC)	34.2
Ceramide aminoethylphosphonate (CAEP)	10.6

**Table 2:** Fatty acid composition of ether phospholipids from scallop (%).

Fatty acid	ePE	ePC
C22 : 6	31.9	43.2
C20 : 5	26.4	9.6
C20 : 4	10.2	3.2
C16 : 1	2.2	4.0
C18 : 1	2.6	3.2
C14 : 0	0.7	9.1
C16 : 0	3.6	15.9
C18 : 0	2.5	6.4
Others	19.8	4.2

ePE; ethanolamine ether phospholipid, ePC; choline ether phospholipid.

**Table 3:** Baseline characteristics of patients with PD.

Variable	Parkinson's disease (*n* = 10), mean	Normal control (*n* = 39), mean	*p* value^*∗*^
Age	67.80 (7.41)	71.87 (5.50)	
Men/women	3/7	13/26	
MMSE	28.56 (2.13)	29.90 (0.31)	

Plasma lipids			
ePE	2.97 (0.76)	4.27 (1.07)	0.001
ePC	4.07 (0.80)	4.33 (0.74)	0.34
SM	29.1 (3.80)	27.99 (3.53)	0.39

Erythrocyte phospholipids			
plsPE	7.67 (0.78)	8.56 (0.94)	0.008
PE	9.71 (0.81)	10.12 (1.14)	0.29
PC	23.25 (1.74)	25.02 (2.79)	0.06
SM	49.99 (3.10)	46.99 (4.85)	0.07
PS	9.34 (1.14)	9.31 (1.30)	0.94

Values are mean (SD) unless otherwise specified. ePE: ethanolamine ether phospholipid; ePC: choline ether phospholipid; SM: sphingomyelin; plsPE: ethanolamine plasmalogen; PE: diacyl ethanolamine glycerophospholipid; PC: diacyl choline glycerophospholipid; PS: diacyl serine glycerophospholipid. ^*∗*^Based on unpaired *t*-test for the between-group differences.

**Table 4:** Increase of erythrocyte plasmalogen in PD after administration of scallop-derived ether phospholipids.

Variable (%)	Before	After 12 weeks	*p* value^*∗*^	After 24 weeks	*p* value^*∗*^
plsPE	7.67 (0.78)	8.43 (1.2)	0.03	8.64 (0.97)	0.01
PE	9.71 (0.81)	10.12 (0.77)	0.32	10.81 (0.95)	0.03
PC	23.25 (1.74)	23.54 (3.29)	0.83	25.41 (1.74)	0.006
SM	49.99 (3.1)	47.87 (5.41)	0.39	44.83 (2.65)	0.004
PS	9.34(1.14)	10.05 (1.08)	0.13	10.33 (0.75)	0.01

Values are mean (SD) unless otherwise specified. plsPE: ethanolamine plasmalogen; PE: diacyl ethanolamine glycerophospholipid; PC: diacyl choline glycerophospholipid; PS: diacyl serine glycerophospholipid; SM: sphingomyelin. ^*∗*^Differences from immediate before trial.

**Table 5:** Improvement of clinical symptoms of Parkinson's disease after oral administration of scallop-derived ether phospholipids.

PDQ-39	Before (*n* = 10), mean scores	After 24 weeks (*n* = 10), mean scores	*p* value^*∗*^
Total	273.5 (145.2)	188.8 (93.8)	0.02
Mobility	50.5 (29.0)	48.8 (22.9)	0.66
Daily activity	48.8 (23.7)	35.4 (16.3)	0.05
Emotional well-being	40.4 (27.5)	26.3 (19.1)	0.07
Stigma	23.1 (17.9)	15.0 (17.7)	0.05
Social support	17.5 (18.2)	6.7 (12.3)	0.03
Cognitions	40.7 (19.8)	25.0 (16.4)	0.02
Communication	35.8 (33.8)	22.5 (26.4)	0.07
Bodily discomfort	16.7 (11.1)	9.2 (10.0)	0.03

Values are mean (SD) unless otherwise specified. ^*∗*^Differences from before trial.

## Data Availability

All the data used to support the findings of this study are included within the article.
